# Expression profile of mRNAs and miRNAs related to mitogen-activated kinases in HaCaT cell culture treated with lipopolysaccharide a and adalimumab

**DOI:** 10.1080/15384101.2024.2335051

**Published:** 2024-04-01

**Authors:** Michał Wójcik, Aleksandra Plata-Babula, Amelia Głowaczewska, Tomasz Sirek, Aneta Orczyk, Mariola Małecka, Beniamin Oskar Grabarek

**Affiliations:** a Collegium Medicum, WSB University, Dabrowa Gornicza, Poland; b Department of Nursing and Maternity, High School of Strategic Planning in Dabrowa Gornicza, Dabrowa Gornicza, Poland; c Faculty of Health Sciences, University of Applied Sciences in Nysa, Nysa, Poland; d Department of Plastic Surgery, Faculty of Medicine, Academia of Silesia, Katowice, Poland; e Department of Plastic and Reconstructive Surgery, Hospital for Minimally Invasive and Reconstructive Surgery in Bielsko-Biała, Bielsko-Biala, Poland; f Faculty of Medicine, Uczelnia Medyczna im. Marii Skłodowskiej-Curie, Warszawa, Poland

**Keywords:** Mitogen-activated protein kinases, adalimumab, lipopolysaccharides

## Abstract

Studies indicate that mitogen-activated protein kinases (MAPKs) exhibit activation and overexpression within psoriatic lesions. This study aimed to investigate alterations in the expression patterns of genes encoding MAPKs and microRNA (miRNA) molecules that potentially regulate their expression in human adult low-calcium high-temperature (HaCaT) keratinocytes when exposed to bacterial lipopolysaccharide A (LPS) and adalimumab. HaCaT cells underwent treatment with 1 µg/mL LPS for 8 hours, followed by treatment with 8 µg/mL adalimumab for 2, 8, or 24 hours. Untreated cells served as controls. The molecular analysis involved microarray, quantitative real-time polymerase chain reaction (RTqPCR), and enzyme-linked immunosorbent assay (ELISA) analyses. Changes in the expression profile of seven mRNAs: dual specificity phosphatase 1 (*DUSP1)*, dual specificity phosphatase 3 (*DUSP3)*, dual specificity phosphatase 4 (*DUSP4)*, mitogen-activated protein kinase 9 (*MAPK9)*, mitogen-activated protein kinase kinase kinase 2 (*MAP3K2)*, mitogen-activated protein kinase kinase 2 (*MAP2K2), and* MAP kinase-activated protein kinase 2 *(MAPKAPK2*, also known as *MK2)* in cell culture exposed to LPS or LPS and the drug compared to the control. It was noted that miR-34a may potentially regulate the activity of *DUSP1*, *DUSP3*, and *DUSP4*, while miR-1275 is implicated in regulating *MAPK9* expression. Additionally, miR-382 and miR-3188 are potential regulators of *DUSP4* levels, and miR-200-5p is involved in regulating *MAPKAPK2* and *MAP3K2* levels. Thus, the analysis showed that these mRNA molecules and the proteins and miRNAs they encode appear to be useful molecular markers for monitoring the efficacy of adalimumab therapy.

## Introduction

Mitogen-activated protein kinases (MAPKs) play a crucial role in the molecular processes underlying psoriasis [1]. They function in transmitting signals within the cell through a series of reactions, leading to the activation of various proteins. Consequently, MAPKs-dependent signaling pathways exert regulatory effects on transcription factors, protein synthesis, cell division, differentiation, and impact cell survival as well as apoptosis [2]. MAPKs can be categorized into three groups: 1) extracellular signal-regulated kinases (ERKs), 2) c-Jun N-terminal kinases (JNKs), and 3) p38 MAPKs [3]. ERK1 and ERK2 represent two isoforms of ERK, participating in molecular pathways that regulate the cell cycle, cell proliferation, and differentiation [4]. JNKs, known as stress-activated kinases, consist of three isoforms: JNK1, JNK2, and JNK3 (unique to the brain and heart) [5]. JNK signaling pathways are activated by stressors like ultraviolet radiation, influencing processes such as cell differentiation, apoptosis, angiogenesis, and migration [5]. In psoriasis, JNK activation in keratinocytes leads to increased production of pro-inflammatory cytokines and chemokines, contributing to the inflammatory milieu of psoriatic lesions [6]. In turn, p38 MAPKs comprise four isoforms and are involved in cell differentiation and apoptosis. Activation of p38 MAPKs occurs in response to stress factors, as well as the action of inflammatory cytokines and growth factors.

Psoriasis, characterized by diverse dermatological manifestations, is a persistent inflammatory autoimmune ailment [7]. Clinical categorization identifies subtypes such as psoriasis vulgaris, inverse psoriasis, guttate psoriasis, pustular psoriasis, and erythrodermic psoriasis. Psoriatic arthritis can also manifest in the joints as a consequence [7]. Despite substantial advancements in psoriasis treatment and increased research on the condition, it remains challenging for both patients and physicians [8]. The exact etiology and pathogenesis of psoriasis are not entirely understood, leading to a lack of precise therapeutic approaches [8]. Identifying the factors contributing to and exacerbating psoriasis is complicated by intricate interactions involving genetic predisposition, environmental elements, and the modulation of immunological processes [9]. During psoriasis exacerbations, clinical changes, including parakeratosis, infiltration of neutrophils, lymphocytes, and cytokines in the epidermis and dermis, occur. These alterations stem from an accelerated abnormal maturation cycle of keratinocytes, coupled with heightened inflammatory infiltration [8]. A distinctive symptom of psoriasis is the occurrence of parakeratosis, indicating an eightfold acceleration in epidermal keratinization [8]. Psoriasis, being an incurable condition, exposes patients to potential side effects of the medications they use [10]. Treatment objectives aim to prolong remission, alleviate symptoms, and enhance the quality of life for psoriasis patients [10]. Medications, with either localized or generalized effects, operate by mechanisms that decelerate keratinocyte metabolism, restrict cell differentiation, and eliminate scales [11,12].

It is crucial to consider that psoriasis keratinocytes display resistance to signals transmitted by TNF-α, resulting in an elevated concentration of TNF-α in skin alterations and serum among psoriasis patients [13,14]. This resistance prompted the use of TNF-α inhibitors in the therapy of moderate and acute psoriasis, with adalimumab being one such inhibitor known for its ability to bind and deactivate both the membrane and soluble forms of TNF-α [15]. As per the 2020 guidelines from the Polish Dermatological Society, adalimumab is endorsed for addressing moderate and severe instances of plaque psoriasis and psoriatic arthritis. According to the recommendations, the dose of adalimumab should be increased gradually. In the first week of treatment, a dose of 80 mg is recommended, followed by 40 mg of the drug in the second week, and then 40 mg every week thereafter [16,17]. Despite an incomplete understanding of psoriasis pathogenesis and the fact that current treatment endeavors do not achieve complete symptom eradication, it is recognized that various external and internal factors influence the development and progression of the disease [13,18].

In psoriasis, p38 MAPK is activated in keratinocytes and immune cells, leading to the production of inflammatory mediators, such as tumor necrosis factor-alpha (TNF-α) and interleukin-17 (IL-17) [14]. Moreover, Zhu et al. suggest that the introduction of a new class of drugs affecting the p38 MAPK pathway may overcome the current failures in the treatment of psoriasis [15].

Research suggests that MAPKs are both activated and overexpressed in psoriatic lesions [19]. The activation of the JNK pathway in keratinocytes is implicated in regulating the production of inflammatory cytokines, thereby affecting the recruitment of immune cells [20]. This mechanism is particularly significant in autoimmune arthritic conditions, including rheumatoid arthritis, ankylosing spondylitis, and psoriatic arthritis [21]. Targeting MAPK pathways has been explored as a therapeutic strategy for psoriasis [20,22]. Inhibitors of MAPK pathways, such as JNK inhibitors, ERK inhibitors, and p38 MAPK inhibitors, are being investigated for their potential to control the inflammatory and proliferative processes associated with psoriasis [23–26].

It is important to note that the understanding of the molecular mechanisms underlying psoriasis is continually evolving, and ongoing research may reveal new insights into the role of MAPKs in this skin disorder [27]. Therapies targeting MAPK pathways represent a promising area for the development of novel psoriasis treatments [23–26].

A literature review conducted by Assefi et al. indicates that the use of MAPK inhibitors may be particularly helpful in the treatment of patients who have developed drug resistance to therapy using TNF-α inhibitors. Although further research is needed to improve the safety profile of MAPK inhibitors [28]. In turn, Xiao et al. suggest that the miR-203-LXR-α/PPAR-γ axis modulates keratinocyte proliferation and may constitute a new target in the treatment of psoriasis [29].

The aim of the study was to assess changes in the expression pattern of genes encoding MAPKs and miRNA molecules potentially regulating their expression in human adult low-calcium high-temperature (HaCaT) keratinocytes exposed to bacterial lipopolysaccharide A (LPS) and adalimumab.

## Material and methods

### Keratinocyte cell culture

The HaCaT culture was conducted in 25 cm^2^ culture vessels and placed in a Direct Heat CO2 incubator (Thermo Fisher Scientific, Waltham, MA) set at 37°C with 5% CO2. The culture medium used was Dulbecco’s modified Eagle’s medium (DMEM; Sigma-Aldrich, St. Louis, MO, USA), supplemented with glucose (4500 mg/L, Sigma-Aldrich, St. Louis, MO, USA), 10% fetal bovine serum (FBS; Sigma-Aldrich, St. Louis, MO, USA), penicillin (100 U/mL; Sigma-Aldrich, St. Louis, MO, USA), streptomycin (100 mg/mL; Sigma-Aldrich, St. Louis, MO, USA), and glutamine (2 mM; Sigma-Aldrich, St. Louis, MO, USA).

In the initial phase, aligning with our prior investigations, HaCaT cells underwent an 8-hour incubation with 1 μg/mL of LPS [30–33] to induce inflammation. Subsequently, adalimumab was introduced at a concentration of 8 μg/mL for 2, 8, and 24 hours in the following stage. The control culture remained untreated with LPS and the drug. The selection of the adalimumab concentration was empirical, considering the average adalimumab concentration in the serum of psoriasis-treated patients, consistent with our previous research [31,32].

### Cytotoxicity assay of LPS and adalimumab

To evaluate the potential cytotoxic effects of LPS and adalimumab on keratinocytes, we conducted an XTT assay using the In Vitro Toxicology Assay Kit (Sigma Aldrich, St Louis, MO, USA), following the manufacturer’s instructions. The XTT assay principle relies on converting a yellow tetrazole salt to an orange formazan by mitochondrial dehydrogenases in metabolically active (living) cells, with the amount of formazan directly proportional to cell viability. To assess the impact of LPS and adalimumab, HaCaT cells were treated with varying concentrations of LPS (1 μg/ml, 2 μg/ml, and 10 μg/ml) to induce inflammation and adalimumab (0.8 μg/ml, 8 μg/ml, and 80 μg/ml). Untreated cells served as the experimental control. The absorbance readings obtained were used to calculate the absorption values for each concentration of LPS and adalimumab relative to the control cells (set at 100%). This allowed us to determine the percentage of viable cells in cultures treated with LPS and adalimumab compared to untreated cells [33].

### Total ribonucleic acid (RNA) isolations

Total RNA isolation from HaCaT cultures exposed to LPS and adalimumab, as well as the control group, was carried out using Trizol reagent (Invitrogen Life Technologies, Carlsbad, CA, USA; Catalog number: 15596026) according to the manufacturer’s guidelines. Subsequently, the RNA extracts obtained were purified using the RNeasy mini kit (QIAGEN, Hilden, Germany; Catalog number: 74104) and DNase I (Fermentas International Inc., Burlington, ON, Canada; Catalog number: 18047019).

A qualitative assessment of the extracts was performed through 1% agarose gel electrophoresis with Simply Safe dye (EurX, Gdańsk, Poland). Electrophoretic separation was carried out using a Submini apparatus (Kucharczyk, Warsaw, Poland). By analyzing the electropherogram under UV transilluminator light and utilizing a computerized gel documentation system, two bands corresponding to the 28S rRNA and 18S rRNA fractions were identified. Simultaneously, the concentration and purity of the extracts were determined using a spectrophotometer (Nanodrop®, Thermo Fisher Scientific, Waltham, MA, USA).

The purity of the RNA isolates was evaluated based on the A260/A280 absorbance ratio, which was expected to fall within the range of 1.80–2.00.

### Assessment of the expression pattern of MAPKs via oligonucleotide microarrays

The expression profile of genes encoding MAPKs was evaluated using HG-U133_A2 oligonucleotide microarrays (Affymetrix, Santa Clara, CA, USA) and the GeneChip™ 3′ IVT PLUS reagent kit (Affymetrix, Santa Clara, CA, USA; Catalog Number 902,416) following the manufacturer’s guidelines. Each reaction was conducted in triplicate, involving five main steps: 1) cDNA synthesis; 2) cRNA synthesis, labeling, and fragmentation; 3) hybridization of samples with probes on the microarray plate; 4) reading the fluorescence signal using the Affymetrix Gene Array Scanner 3000 7 G and Gene Chip® Command Console® Software (Affymetrix, Santa Clara, CA, USA); 5) data analysis.

On the HG-U133A_2 microarray plate, there are 22,277 mRNA probes with 3 degrees of complementarity to the target transcript, namely “_s_at”. This identifier format typically represents probes that correspond to a unique gene transcript. The “_s” designation usually stands for “single” probe, indicating that it is designed to target a single-gene transcript with high specificity. These probes are intended to be specific to a particular transcript sequence.

“x_at” probes, on the other hand, often refer to probes that may map to multiple transcripts or exhibit some level of ambiguity in their target specificity. The “x” designation typically denotes a “cross-mapping” probe, suggesting that it can map to multiple regions of the genome or to multiple transcripts. While these probes may offer broader coverage, they can also introduce greater ambiguity in interpretation.

Probes labeled “_at” are designed to target a specific region of the genome rather than a specific transcript. The “_at” designation is often used for probes that are not explicitly assigned to a transcript but rather to a genomic locus. These probes may cover a region that contains multiple transcripts or may not be associated with any known transcript.

In summary, the different probe identifier formats serve distinct purposes in gene expression microarray experiments, allowing researchers to tailor their analyses based on their specific needs for transcript specificity or genomic coverage.

The list of genes related to MAPK signaling pathways was prepared based on data from the PathCards database (http://pathcards.genecards.org/) accessed on December 1, 2023 [34].

### Predicted analysis of miRnas regulating mRNA MAPKs pattern via miRNA microarrays

The examination of miRNAs displaying significant changes in expression in HaCaT culture exposed to LPS and adalimumab was conducted using GeneChip miRNA 2.0 arrays (Affymetrix, Santa Clara, CA, USA), following the manufacturer’s guidelines. To assess the potential impact of the identified miRNAs on the expression of MAPKs genes, the Targetscan database (http://www.targetscan.org/; accessed December 3, 2023) [35] and miRanda (http://mirdb.org; accessed December 3, 2023) were employed [36]. A predicted target with a prediction score exceeding 80 is deemed highly likely to be authentic. However, caution is advised if the score falls below 60, and it is recommended to seek additional supporting evidence [36,37].

### Reverse transcription-quantitative polymerase chain reaction (RT-qPCR)

The outcomes obtained from the microarray experiment were validated using RT-qPCR. The RT-qPCR analysis targeted specific genes, including dual specificity phosphatase 1 (*DUSP1*), dual specificity phosphatase 3 (*DUSP3*), dual specificity phosphatase 4 (*DUSP4*), mitogen-activated protein kinase kinase 2 (*MAP2K2*), mitogen-activated protein kinase kinase 7 (*MAP2K7*), mitogen-activated protein kinase kinase kinase 2 (*MAP3K2*), mitogen-activated protein kinase 9 (*MAPK9*), and beta-actin (*ACTB*) served as an endogenous control. Utilizing the Sensi-Fast reagent kit (Bioline, London, England) and the primers listed in Table 1, reverse transcription and PCR were performed in the same tube without altering the reaction mixture, in a total volume of 50 μL.

**Table 1.** Nucleotide sequence of primers used in RT-qPCR.

mRNA	Starter	Sequence	Product size (bp)	Tm (°C)
*DUSP1*	Forward	5’- GGATACGAAGCGTTTTCGGC-3’	1531	85.5
	Reverse	5’-AGAGGTCGTAATGGGGCTCT-3’		
*DUSP3*	Forward	5’-GAGATTCCCACAGCAAGGCT-3’	219	79.9
	Reverse	5’-GCCCAAGGCATCACTCTTCT-3’		
*DUSP4*	Forward	5’-GACCGGCAAAAATACACGGG-3’	520	91.6
	Reverse	5’-GAACCTAGGATGTAGCCCGC-3’		
*MAP2K2*	Forward	5’- AGCTGGAGGAGCTGGAACTTG-3’	817	89.5
	Reverse	5’- CTATCCATCCCGTGACCGC-3’		
*MAPKAPK2*	Forward	5’-AGTATGACAAGTCCTGTGAC-3’	183	84.4
	Reverse	5’-TCTTCACTTCCTCTGATACTTC-3’		
*MAP3K2*	Forward	5’-TCTGTTTTATCTTCTCAGGCCA-3’	958	80.5
	Reverse	5’- CCCTGGGTCCTTCTAGCTCT-3’		
*MAPK9*	Forward	5’-AGTCATCCTGGGTATGGGCT-3’	570	81.3
	Reverse	5’-GCGTTGCTACTTACTGCTGC-3’		
*ACTB*	Forward	5’-TCACCCACACTGTGCCCATCTACGA-3’	295	87.8
	Reverse	5’-CAGCGGAACCGCTCATTGCCAATGG-3’		

*DUSP1* – dual specificity phosphatase 1; *DUSP3* – dual specificity phosphatase 3; *DUSP4* – dual specificity phosphatase 4; *MAPK9 -* mitogen-activated protein kinase 9; *MAP3K2 -* mitogen-activated protein kinase kinase kinase 2; *MAP2K2 -* mitogen-activated protein kinase kinase 2; *MAPKAPK2 -* MAP kinase-activated protein kinase 2ACTB, beta actin; bp, base pair; Tm, melting temperature.

An additional and negative control was also performed. As a positive control, we used an absolute standard, a nucleic acid matrix with a known copy number that provides quantitative information. For the negative control, instead of extracted RNA, we added deionized water to the reaction mixture.

Simultaneously with the test samples, an RT-qPCR reaction for *ACTB* mRNA was conducted to validate the integrity of the RNA extracts and assess the accuracy of the amplification run. Each reaction was conducted in triplicate. The thermal profile of the reaction included: 1) reverse transcription (45°C; 10 min); 2) initial denaturation (95°C; 2 min); 3) 40 cycles comprising denaturation (95°C; 5 s), primer annealing (60°C; 10 s), and primer elongation (72°C; 5 s). The results were expressed using the 2^−∆∆Ct^ method, where a fold change equal to 1 represented the control, >1 indicated overexpression, and <1 indicated silencing. The specificity of the RT-qPCR reaction was confirmed through 6% polyacrylamide gel electrophoresis and melting curve analysis.

### Evaluation of DUSP1, DUSP4, MAP2K2, MAP2K7, MAPK9 concentration by Enzyme-Linked Immunosorbent Assay (ELISA)

In the initial step, the HaCaT culture underwent washing with cold phosphate-buffered saline (PBS) and was subsequently lysed using radioimmunoprecipitation assay lysis buffer (0.5% deoxycholate, 1% Nonidet *p*-40, 0.1% sodium dodecyl sulfate, 100 μg/mL phenylmethylsulfonyl fluoride, 1 mM Na_2_VO_4_, and 8.5 μg/mL aprotinin in PBS) with shaking for 20 minutes at 4°C. This process facilitated the extraction of total protein from the cells. Following this, samples were collected using a scraper, incubated for 60 minutes at 4°C, and then centrifuged for 15 minutes at 4°C. In the subsequent step, the samples were transferred to ELISA plate wells coated with biotin-labeled antibodies, washed, and incubated with the avidin-horseradish peroxidase complex. To create a standard curve, the average optical density and concentration of each standard were plotted, and the result was multiplied by the dilution factor. Protein concentrations of DUSP1 (MyBioSource, Inc. San Diego, CA, USA; Cat no. MBS761028), DUSP3 (MyBioSource, Inc.San Diego, CA 92,195–3308 USA; Cat no MBS2887773), DUSP4 (MyBioSource, Inc. San Diego, CA, USA; Cat. no. MBS2885922), MAP2K2 (MyBioSource, Inc. San Diego, CA, USA; Cat no MBS9331134), MAPKAPK2 (MyBioSource, Inc. San Diego, CA, USA; Cat no MBS2602774), MAP3K2 (MyBioSource, Inc. San Diego, CA, USA; Cat no MBS9339652), and MAPK9 (MyBioSource, Inc. San Diego, CA, USA; Cat no MBS1602553) were evaluated by ELISA in accordance with the manufacturer’s instructions. The assay included both positive controls, utilizing cells derived from the human cervical cancer cell line (HeLa), and negative controls, which consisted of samples lacking the primary antibody were provided. Each reaction was carried out in triplicate.

### Statistical analysis

The acquired data underwent statistical analysis using Transcriptome Analysis Console (Thermo Fisher, USA) and STATISTICA 13.5 PL (StatSoft, Cracow, Poland) software, with a statistical significance threshold set at *p* < 0.05. The analysis involved assessing result conformity with a normal distribution, determined by the Shapiro–Wilk test. Following confirmation of normal distribution assumptions, parametric tests, such as one-way analysis of variance (ANOVA) with Bonferroni correction, were applied. Prior to ANOVA, Levene’s test was conducted, and subsequent to ANOVA, either Tukey’s post hoc test or Student’s t-test was employed.

The relationships between genes were also analyzed in detail using the Search Tool for the Retrieval of Interacting Genes/Proteins (String Database 11.0; accessed January 10, 2024) [38]. For the STRING database, the parameter strength Log10 (observed/expected) describes the magnitude of the enrichment effect. It represents the ratio between i) the number of proteins in the network annotated with a specific term and ii) the number of proteins we would expect to be annotated with that term in a randomly generated network of the same size. Conversely, the parameter false discovery rate describes the significance of the enrichment. Presented are p-values adjusted for multiple testing within each category using the Benjamini – Hochberg procedure [38].

## Results

### Changes in MAP kinase mRNA expression obtained by microarray experiment

**Figure 1.**
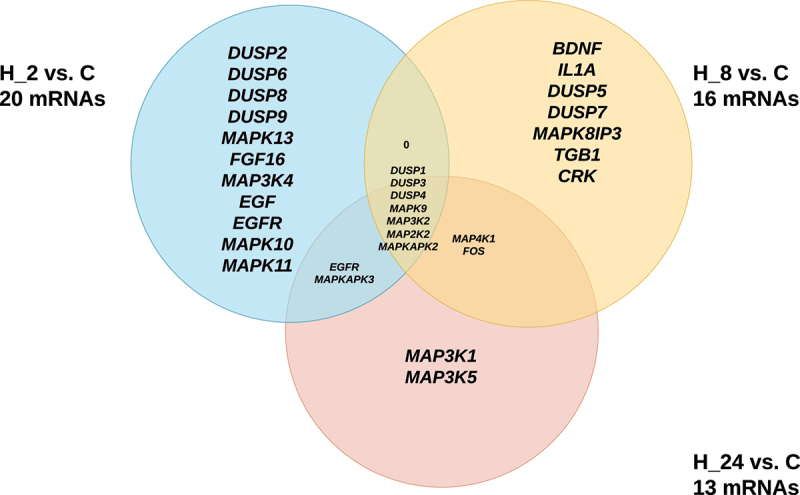
The Venn diagram of microarray results.

Out of 244 mRNAs related to MAPK-signaling pathways, according to the ANOVA with Bonferroni correction significant expression changes between HaCaT culture exposed to LPS and adalimumab from the control culture were observed for 51 mRNAs, including H_2 vs. C = 20 mRNAs, H_8 vs. C = 16 mRNAs, H_24 vs. C = 13 mRNAs. In turn, 7 mRNAs differentiating the culture with the drug regardless of the incubation time: dual specificity phosphatase 1 (*DUSP1)*, dual specificity phosphatase 3 (*DUSP3)*, dual specificity phosphatase 4 (*DUSP4)*, mitogen-activated protein kinase 9 (*MAPK9)*, mitogen-activated protein kinase kinase kinase 2 (*MAP3K2)*, mitogen-activated protein kinase kinase 2 (*MAP2K2), and* MAP kinase-activated protein kinase 2 *(MAPKAPK2*, also known as *MK2)*. The expression pattern of these 7 genes in HaCaT culture exposed to LPS or LPS and the adalimumab compared to the control are presented in Table 2. We observed that the introduction of LPS to the HaCaT culture resulted in the suppression of expression for all transcripts. Conversely, upon the addition of the drug to the cell culture, we observed an augmentation in the transcriptional activity of all 7 transcripts. Further analysis using Tukey’s post hoc test and a Venn diagram unveiled genes that were unique to specific incubation times with LPS and adalimumab, as well as those common across multiple groups (Figure 1).

**Table 2.** Expression pattern of MAPK-related genes in the HaCaT cell line exposed to LPS and then adalimumab for 2, 8, 24 hours compared to the control (*p* < 0.05).

		LPS	LPS+adalimumab		
		log_2_ Fold Change			
ID	mRNA	H_8 vs C	H-2 vs C	H-8 vs C	H-24 vs C
201041_s_at	*DUSP1*	(−)2.96 ± 0.34	(+)7.98 ± 0.37	(+)6.53 ± 0.55	(+)3.07 ± 0.13
201044_x_at		(−)3.02 ± 0.19	(+)6.26 ± 0.41	(+)5.84 ± 0.65	(+)4.98 ± 0.87
201536_at	*DUSP3*	(−)1.45 ± 0.14	(+)1.97 ± 0.54	(+)1.44 ± 0.31	(+)1.51 ± 0.13
201537_s_at		(−)1.71 ± 0.17	(+)2.11 ± 0.24	(+)1.51 ± 0.41	(+)1.11 ± 0.51
204014_at	*DUSP4*	(−)2.19 ± 0.76	(+)4.42 ± 0.24	(+)1.08 ± 0.11	(+)1.02 ± 0.18
204015_s_at		(−)2.77 ± 0.81	(+)2.88 ± 0.62	(+)1.04 ± 0.18	(+)1.05 ± 0.09
213490_s_at	*MAP2K2*	(−)3.51 ± 0.13	(+)2.21 ± 0.34	(+)3.13 ± 0.12	(+)3.54 ± 0.86
215050_x_at	*MAPKAPK2*	(−)4.70 ± 0.16	(+)2.31 ± 0.12	(+)2.17 ± 0.34	(+)3.50 ± 0.14
221695_s_at	*MAP3K2*	(−)4.11 ± 0.22	(+)3.18 ± 0.34	(+)3.87 ± 0.61	(+)3.81 ± 0.12
210570_x_at	*MAPK9*	(−)4.71 ± 0.31	(+)3.12 ± 0.55	(+)3.16 ± 0.34	(+)2.21 ± 0.11
203218_at		(−)4.21 ± 0.18	(+)3.45 ± 0.81	(+)3.19 ± 0.21	(+)1.89 ± 0.24

(+) – overexpression compared to the control; (−) – downregulated compared to the control; LPS – lipopolysaccharide A; C – control culture; H_2, H_8, H_24 – culture exposed to adalimumab for 2, 8, 24 hours; DUSP1 – dual specificity phosphatase 1; DUSP4 – dual specificity phosphatase 4; MAP2K2 – mitogen-activated protein kinase kinase 2; MAP2K7 – mitogen-activated protein kinase kinase 7; MAP3K2 – mitogen-activated protein kinase kinase kinase 2; MAPK9 – mitogen-activated protein kinase 9.

C – control culture; H_2, H_8, H_24 – culture exposed to adalimumab for 2, 8, 24 hours; *DUSP1* – dual specificity phosphatase 1; *DUSP3* – dual specificity phosphatase 3; *DUSP4* – dual specificity phosphatase 4; *MAPK9 -* mitogen-activated protein kinase 9; *MAP3K2 -* mitogen-activated protein kinase kinase kinase 2; *MAP2K2 -* mitogen-activated protein kinase kinase 2; *MAPKAPK2 -* MAP kinase-activated protein kinase 2

### Changes in MAPKs mRNA expression obtained by RT-qPCR

**Figure 2.**
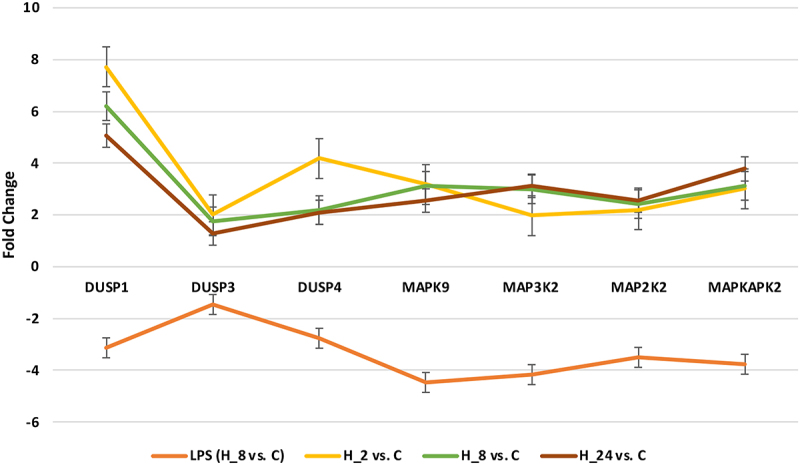
The mRNA expression profile of chosen MAPK-related genes in HaCaT cells was examined after treatment with LPS followed by adalimumab a for durations of 2, 8, and 24 hours, in comparison to the control culture.

We confirmed the microarray experiment by employing RT-qPCR to analyze 7 specific mRNAs, distinguishing keratinocyte cultures from LPS and adalimumab, irrespective of the duration of drug exposure (Figure 2). Consistently, we observed a concordant direction of expression change in both the microarray and RT-qPCR analyses.

LPS – lipopolysaccharide A; C – control culture; H_2, H_8, H_24 – culture exposed to adalimumab for 2, 8, 24 hours; DUSP1 – dual specificity phosphatase 1; DUSP3 – dual specificity phosphatase 3; DUSP4 – dual specificity phosphatase 4; *MAPK9 -* mitogen-activated protein kinase 9; *MAP3K2 -* mitogen-activated protein kinase kinase kinase 2; *MAP2K2 -* mitogen-activated protein kinase kinase 2; *MAPKAPK2 -* MAP kinase-activated protein kinase 2

The statistical analysis revealed a significant increase in the expression of all seven mRNAs in the keratinocyte culture treated with both LPS and adalimumab (across all durations of adalimumab treatment) compared to the culture exposed to LPS alone (one-way analysis of variance ANOVA; *p* < 0.001). In addition, in Table 3 we present the *p*-values for the ANOVA and Tukey’s post-hoc. Therefore, Tukey’s post hoc test revealed statistically significant differences in the expression of each of the 7 analyzed transcripts between cultures exposed to LPS and cultures to which the drug was added, irrespective of the incubation time of the cells with adalimumab (Table 3; *p* < 0.05). Conversely, the variations in the expression of the 7 analyzed genes based on the incubation time of keratinocyte cultures with adalimumab were mostly not statistically significant (Table 3; *p* > 0.05).

**Table 3.** Results of ANOVA and Tukey’s post-hoc test for MAPK genes, the expression of which was determined by the qRT-PCR technique.

		Adjusted *p*-value of Tukey’s post-hoc test					
mRNA	*p*-value ANOVA	LPS vs. H_2	LPS vs. H_8	LPS vs. H_24	H_2 vs. H_8	H_2 vs. H_24	H_8 vs. H_24
*DUSP1*	<0.001	<0.001	<0.001	<0.001	0.043	<0.001	<0.001
*DUSP3*	<0.001	<0.001	<0.001	<0.001	0.761	<0.001	0.501
*DUSP4*	<0.001	<0.001	<0.001	<0.001	<0.001	<0.001	0.879
*MAP2K2*	<0.001	<0.001	<0.001	<0.001	0.021	0.019	0.163
*MAPKAPK2*	<0.001	<0.001	<0.001	<0.001	0.287	0.027	0.021
*MAP3K2*	<0.001	<0.001	<0.001	<0.001	0.871	0.765	0.900
*MAPK9*	<0.001	<0.001	<0.001	<0.001	0.419	<0.001	0.041

LPS – lipopolysaccharide A; C – control culture; H_2, H_8, H_24 – culture exposed to adalimumab for 2, 8, 24 hours; DUSP1 – dual specificity phosphatase 1; DUSP4 – dual specificity phosphatase 4; MAP2K2 – mitogen-activated protein kinase kinase 2; MAP2K7 – mitogen-activated protein kinase kinase 7; MAP3K2 – mitogen-activated protein kinase kinase kinase 2; MAPK9 – mitogen-activated protein kinase 9.

### miRNA expression

In the subsequent phase, we identified specific miRNAs that differentiate the keratinocyte culture exposed to the drug from the control culture, considering the miRSVR parameter, and that potentially modulate the expression of *DUSP1*, *DUSP3*, *DUSP4*, *MAPK9*, *MAP3K2*, *MAP2K2*, and *MAPKAPK2*.

Our findings indicate that miR-34a has the potential to regulate the activity of *DUSP1*, *DUSP3*, and *DUSP4*. Additionally, miR-1275 is implicated in the regulation of *MAPK9* expression. Furthermore, miR-382 and miR-3188 are potential regulators of *DUSP4* levels, and miR-200-5p is involved in regulating *MAPKAPK2* and *MAP3K2* levels. However, according to predictive analysis, no regulatory effect of miRNA molecules on *MAP2K2* expression was observed.

In turn, Table 4 presents changes in the expression of selected miRNAs with statistical significance values obtained in the ANOVA and Tukey’s post-hoc tests. However, the conducted statistical analysis did not reveal a statistically significant change in miR-200a-5p expression in LPS-exposed cultures compared to keratinocyte cultures exposed to adalimumab (one-way ANOVA; *p* > 0.05). For the other selected miRNAs, changes in their expression were deemed statistically significant (one-way ANOVA; *p* < 0.05). Notably, only miR-200a-5p exhibited a reduction in expression in LPS-exposed HaCaT cultures compared to cultures not treated with the pro-inflammatory agent (Table 4; *p* < 0.05).

**Table 4.** Expression pattern of miRNAs potentially regulating the expression of *DUSP1*, *DUSP3, DUSP4*, *MAPK9, MAP3K2, MAP2K2*, *MAPKAPK2* and of the HaCaT cell line exposed to LPS and then adalimumab for 2, 8, 24 hours compared to the control.

		Expression					
mRNA regulated by selected miRNA	miRNA	LPS vs. C	H-2 vs C	H-8 vs C	H-24 vs C	*p*-value ANOVA	*p*-value Tukey’s post-hoc test
*DUSP1* *DUSP3 DUSP4*	miR-34a	(+)6.41 ± 0.14	(+)4.15 ± 0.51	(+)3.84 ± 0.41	(+)3.21 ± 0.10	<0.001	<0.001^1,2,3^ 0.048^4^ 0.031 ^5^ 0.087^6^
*MAPK9*	miR-1275	(+)4.12 ± 1.11	(-)1.84 ± 0.07	(-)2.29 ± 0.32	(-)2.55 ± 0.81	<0.001	<0.001^1,2,3,4^ 0.659^5^ 0.456^6^
*DUSP4*	miR-3188	(+)2.07 ± 0.15	(+)1.74 ± 0.76	(+)2.04 ± 0.19	(+)2.02 ± 0.19	0.019	0.002^1^ 0.898^2^ 0.871 ^3^ 0.113^4^ 0.118^5^ 0.871 ^6^
*DUSP4*	miR-382	(-)2.18 ± 0.29	(+)1.84 ± 0.18	(+)4.29 ± 0.43	(+)3.77 ± 0.23	0.028	<0.001^1,2,3^ 0.419^4^ 0.410^5^ 0.0381^6^
*MAPKAPK2, MAP3K2*	miR-200a-5p	(+)3.09 ± 0.21	(+)2.89 ± 0.54	(+)2.19 ± 0.10	(+)2.11 ± 0.33	0.087	0.478^1^ 0.319^2^ 0.298 ^3^ 0.691^4^ 0.871^5^ 0.893^6^

(+) – overexpression in comparison to the control; (−) – downregulated in comparison to the control; LPS – lipopolysaccharide A; C – control culture; H_2, H_8, H_24 – culture exposed to adalimumab for 2, 8, 24 hours; DUSP1 – dual specificity phosphatase 1; DUSP4 – dual specificity phosphatase 4; *MAPK9 -* mitogen-activated protein kinase 9; *MAP3K2 -* mitogen-activated protein kinase kinase kinase 2; *MAP2K2 -* mitogen-activated protein kinase kinase 2; *MAPKAPK2 -* MAP kinase-activated protein kinase 2; statistically significant differences between: ^1^ LPS vs. H_2; ^2^ LPS vs. H_8; ^3^ LPS vs. H_24; ^4^ H_2 vs. H_8; ^5^ H_2 vs. H_24; ^4^ H_8 vs. H_24.

### Protein concentrations of DUSP1, DUSP3, DUSP4, MAP2K2, MAPKAPK2, MAP3K2, MAPK9

The assessment of the concentration profile of selected proteins related to MAPK pathways showed that the addition of LPS to the HaCaT culture results in a decrease in the expression of each protein compared to the control culture (Table 5; *p* < 0.05). However, for DUSP1, DUSP3, DUSP4, we found higher concentrations in culture with the drug compared to control and culture with LPS (Table 5; *p* < 0.05). On the other hand, for MAP2K2, MAPKAPK2, MAP3K2, MAPK9, the addition of adalimumab to HaCaT cultures resulted in a decrease in their concentration in the culture medium (Table 5; *p* < 0.05).

**Table 5.** Concentration of MAP kinase-related genes at the protein level in HaCaT culture exposed to LPS and adalimumab.

Protein	C [ng/mL]	LPS [ng/mL]	H-2 [ng/mL]	H-8 [ng/mL]	H-24 [ng/mL]	*p*-value Student’s t-test^a^ or ANOVA^b^	*p*-value Tukey’s post-hoc test
DUSP1	0.92 ± 0.11	0.257 ± 0.12	3.76 ± 0.41	3.78 ± 0.19	2.98 ± 0.43	0.002^b^	<0.001^1,2,3,4,5,6,7^ 0.912^8^ 0.703^9^ 0.211 ^10^
DUSP3	1.98 ± 0.32	1.08 ± 0.12	6.45 ± 0.13	6.87 ± 0.12	6.23 ± 0.16	<0.002^b^	<0.001^1,2,3,4,5,6,7^ 0.872^8^ 0.918^9^ 0.891 ^10^
DUSP4	1.06 ± 0.11	0.56 ± 0.01	2.81 ± 0.54	3.01 ± 0.15	2.11 ± 0.38	<0.002^b^	<0.001^1,2,3,4,5,6,7^ 0.231^8^ 0.345^9^ 0.761^10^
MAP2K2	2.11 ± 0.12	1.87 ± 0.34	0.28 ± 0.06	0.20 ± 0.08	0.17 ± 0.05	<0.003^b^	<0.001^1,2,3,4,5,6,7^ 0.851^8^ 0.879^9^ 0.871^10^
MAPKAPK2	1.79 ± 0.19	1.56 ± 0.12	0.98 ± 0.12	0.86 ± 0.09	0.87 ± 0.09	<0.002^b^	<0.001^1,2,3,4,5,6,7^ 0.876^8^ 0.910^9^ 0.998^10^
MAP3K2	0.98 ± 0.07	0.54 ± 0.05	below the detection threshold	below the detection threshold	below the detection threshold	<0.006^a^	-
MAPK9	4.12 ± 0.78	2.19 ± 0.34	below the detection threshold	below the detection threshold	below the detection threshold	<0.001^a^	-

LPS – lipopolysaccharide A; C – control culture; H_2, H_8, H_24 – culture exposed to adalimumab for 2, 8, 24 hours; LPS – lipopolysaccharide A; C – control culture; H_2, H_8, H_24 – culture exposed to adalimumab for 2, 8, 24 hours; DUSP1 – dual specificity phosphatase 1; DUSP3 – dual specificity phosphatase 3; DUSP4 – dual specificity phosphatase 4; *MAPK9 -* mitogen-activated protein kinase 9; *MAP3K2 -* mitogen-activated protein kinase kinase kinase 2; *MAP2K2 -* mitogen-activated protein kinase kinase 2; *MAPKAPK2 -* MAP kinase-activated protein kinase 2; statistically significant differences between: ^1^C vs. LPS; ^2^C vs. H_2; ^3^C vs. H_8; ^4^C vs. H_24; ^5^LPS vs. H_2; ^6^LPS vs. H_8; ^7^LPS vs. H_24; ^8^H_2 vs. H_8; ^9^H_2 vs. H_24; ^10^H_8 vs. H_24.

### Results of the XTT cytotoxicity assay

The results from the XTT cytotoxicity assay did not reveal any significant impact on the vitality of keratinocytes treated with LPS, adalimumab, or a combination. However, statistical analysis demonstrated notable differences between HaCaT cells exposed to 80 µg/mL adalimumab and the untreated control cells (*p* < 0.05). Specifically, when the highest adalimumab concentration was applied, only 40.64% of cells remained viable compared to the control culture (*p* < 0.05). The detailed results of the XTT test are shown in Table 6.

**Table 6.** Results of the XTT cytotoxicity assay.

Compound	Concentration	Cell viability (% of a control)	p-value of the ANOVA test
LPS	1 µg/mL	98.41 ± 0.99	0.993
	2 µg/mL	98.25 ± 0.94	
	10 µg/L	98.77 ± 0.96	
adalimumab	0.8 µg/mL	97.48 ± 0.97	0.041
	8 µg/mL	98.01 ± 1.01	
	80 µg/mL	40.64 ± 1.32	
LPS + adalimumab	LPS (1 µg/mL) + adalimumab (8 µg/mL)	97.03 ± 0.98	–

LPS, lipopolysaccharide A.

### Evaluation of the relationship and its nature between proteins encoded by genes differentiating HaCaT culture with LPS and drug independently of the time of exposure of cells to the drug

The relationships and their nature between MAP kinase pathway gene products indicated by the overrepresentation test were also analyzed in detail using the STRING database (String Database 11.0). In the aforementioned database, the proteins encoded by the analyzed genes form a tightly linked network of probable protein–protein interactions consisting of 8 edges and 7 nodes (*p* < 0.001; average local clustering coefficient 0.79, average node degree: 2.29). Edges represent interactions between proteins, and the weight of an edge indicates the probability of interaction between proteins. Edges in this context signify protein–protein associations, indicating that they represent specific and meaningful connections. In other words, these proteins collaboratively contribute to a shared function, although it does not necessarily imply that they are physically binding to each other.

**Figure 3.**
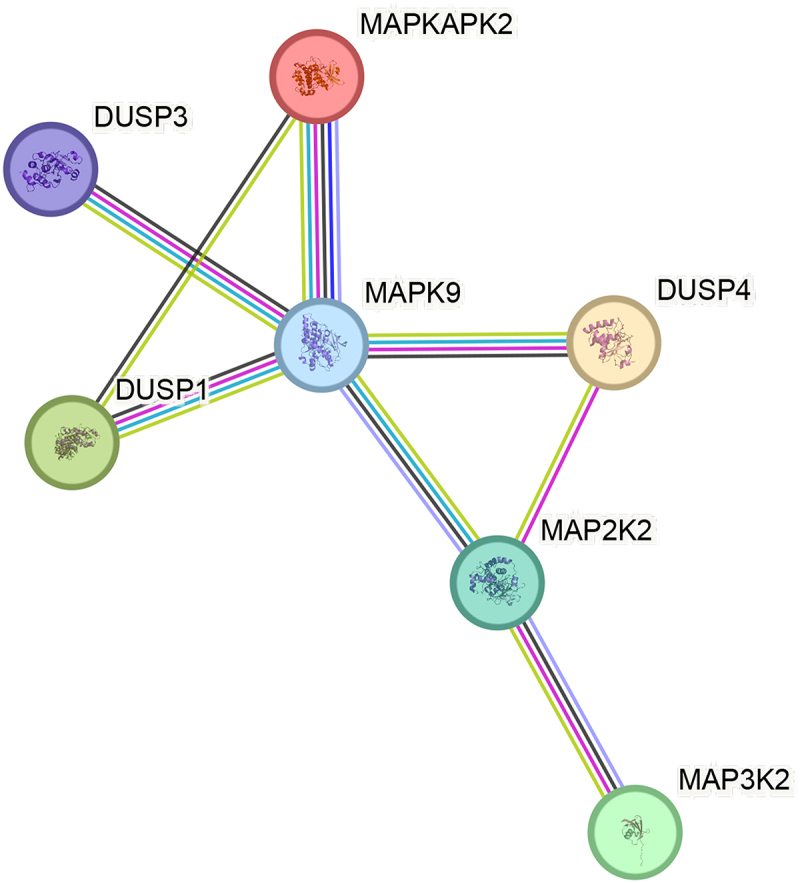
Relationship network for the selected MAPks pathway differentiation genes generated in the STRING database.

The results are depicted in a diagram illustrating interactions between proteins (Figure 3). Furthermore, the analysis conducted segregated the genes encoded by the selected participants into 4 biological processes, 12 molecular functions, and 32 KEGG pathways (Table 7).

**Table 7.** Biologicals and molecular processes, signaling pathways, and protein domains and features of proteins encoded by genes selected in the microarray experiment.

Functional enrichment		Strength	False discovery rate
Biological process	MAPK cascade	1.71	0.0028
	Negative regulation of MAPK cascade	1.70	0.0491
	Phosphate-containing compound metabolic process	1.02	0.0011
	Protein modification process	0.87	0.0034
Molecular function (gene ontology)	Protein tyrosine/threonine phosphatase activity	2.75	0.0044
	MAP kinase phosphatase activity	2.67	0.00018
	MAP kinase tyrosine /serine/threonine phosphatase activity	2.64	0.0056
	Mitogen-activated protein kinase binding	2.32	0.0166
	Protein tyrosine/serine/threonine phosphatase activity	2.27	0.00078
	Protein serine/threonine/tyrosine kinase activity	2.11	0.0288
	Myosin phosphatase activity	2.01	0.0034
	Protein tyrosine phosphatase activity	1.93	0.0039
	Protein serine kinase activity	1.49	0.0038
	Protein serine/threonine kinase activity	1.41	0.0044
	Protein kinase binding	1.21	0.0175
	Catalytic activity, acting on a protein	0.94	0.00069
KEEG pathways	VEGF signaling pathway	2.00	0.0119
	GnRH signaling pathway	1.99	0.00053
	Fc epsilon RI signaling pathway	1.94	0.0132
	Prolactin signaling pathway	1.92	0.00075
	Neurotrophin signaling pathway	1.88	5.03e-11
	MAPK signaling pathway	1.84	0.0152
	Colorectal cancer	1.84	0.0152
	ErbB signaling pathway	1.84	0.0152
	Gap junction	1.81	0.0152
	Endocrine resistance	1.78	0.0152
	Choline metabolism in cancer	1.77	0.0152
	T cell receptor signaling pathway	1.75	0.0152
	C-type lectin receptor signaling pathway	1.75	0.0152
	Toll-like receptor signaling pathway	1.75	0.0152
	Sphingolipid signaling pathway	1.69	0.0154
	Growth hormone synthesis, secretion and action	1.68	0.0154
	Yersinia infection	1.66	0.0156
	Kaposi sarcoma-associated herpesvirus infection	1.65	0.0025
	Relaxin signaling pathway	1.85	0.0156
	FoxO signaling pathway	1.65	0.0156
	Fluid shear stress and atherosclerosis	1.64	0.0156
	Insulin signaling pathway	1.63	0.0156
	Apoptosis	1.63	0.0156
	Autophagy	1.63	0.0156
	Cellular senescence	1.57	0.0173
	Hepatitis B	1.55	0.0272
	Human immunodeficiency virus 1 infection	1.44	0.0272
	Human T-cell lekuemia virus 1 infection	1.43	0.0272
	Salmonella infection	1.43	0.0272
	cAMP signaling pathway	1.43	0.0272
	Ras signaling pathway	1.40	0.0289
	Parkinson disease	1.38	0.0308
Protein domains and features (interPro)	MAP kinase phosphatase	2.75	0.0085
	Rhodanese-like domain superfamily	2.37	0.0279
	Dual specificity phosphatase, catalytic domain	2.35	0.0021
	Rhodanese-like domain	2.35	0.0279
	Dual specificity protein phosphatase domain	2.31	0.0021

DUSP1 – dual specificity phosphatase 1; DUSP3 – dual specificity phosphatase 3; DUSP4 – dual specificity phosphatase 4; *MAPK9 -* mitogen-activated protein kinase 9; *MAP3K2 -* mitogen-activated protein kinase kinase kinase 2; *MAP2K2 -* mitogen-activated protein kinase kinase 2; *MAPKAPK2 -* MAP kinase-activated protein kinase; green line for interaction shows “textmining”; black line for interaction shows “co-expression”; turquoise line for interaction shows “interactions from curated databases”; pink line for interaction shows “interactions experimentally determined”; blue line for interaction shows “gene co-occurrence”; violet line for interaction shows “protein homology”

## Discussion

Modern therapies for conditions characterized by abnormal and uncontrollable expression of cytokines, chemokines, or growth factors aim to restore normal transduction along signaling pathways. Advances in molecular biology and personalized medicine have significantly enhanced our understanding of the molecular basis of many conditions. This progress has enabled the development and ongoing introduction of targeted anti-cytokine treatments [39–41]. An illustrative example of such conditions, where anti-cytokine drugs have been employed for an extended period, includes psoriasis, specifically two of its variants: psoriasis vulgaris and psoriatic arthritis [42–44]. The complexity of its phenotypic heterogeneity poses a significant challenge in determining the expression of genes associated with its development. Therefore, the underlying cause and molecular mechanisms driving psoriasis remain not fully elucidated [45–48]. Biological drugs represent the primary means of managing moderate to severe psoriasis, enhancing the clinical condition for most patients and improving their quality of life, enabling everyday functionality [49–51]. However, the drawbacks of biological treatment include potential drug side effects and the risk of developing resistance to the administered drug [49–51]. In this study, we assessed the impact of a monoclonal anti-TNF-α antibody (adalimumab) on the mRNAs and miRNAs associated with MAPKs-dependent signaling pathways. The rationale behind this investigation lies in the TNF-α-dependent signaling pathways, particularly the MAPKs group of proteins [52,53]. These proteins govern various intracellular processes, including gene transcription, protein biosynthesis, cell division, differentiation, and the regulation of survival or apoptosis. In the context of psoriasis, MAPKs have demonstrated regulatory effects on the expression of genes responsible for pro-inflammatory cytokines [54]. Furthermore, our study built upon the research conducted by Krawczyk et al. [55] and Kjellerup et al. [56]. In our investigation, we assessed alterations in the gene expression of MAPKs pathway influenced by LPS and adalimumab in the HaCaT human keratinocyte cell line.

One of the more recent studies on selected MAPKs pathway transcripts conducted on a group of Polish patients with psoriatic arthritis treated with adalimumab is that of Krawczyk et al. These authors reported a statistically significant decrease in the expression of genes encoding MAPKs family proteins associated with apoptosis [55]. Notably, the HG-U133A 2.0 oligonucleotide microarray technique, identical to the one employed in our study, was utilized by Krawczyk et al. [55]. In turn, Tan et al. observed a statistically significant reduction in the expression of MAPK genes through sequencing [57]. Their findings underscored the considerable participation of these genes in responding to pro-inflammatory cytokines in both psoriasis and rheumatoid arthritis [57].

We observed statistically significant variations in the expression profiles of 7 mRNAs (*DUSP1, DUSP3, DUSP4, MAPK9, MAP3K2, MAP2K2, MAPKAPK2*) in the HaCaT cell culture exposed to LPS and adalimumab, compared to the control culture, irrespective of the incubation time with the drug. When LPS was introduced to the keratinocyte cultures, all transcript expressions were silenced. However, upon the addition of adalimumab to the culture, there was an observed increase in the transcriptional activity of all genes. Subsequently, we demonstrated that miR-34a potentially regulates the activity of *DUSP1*, *DUSP3*, and *DUSP4*; miR-1275 is involved in the regulation of *MAPK9* expression; miR-382 and miR-3188 potentially regulate *DUSP4* levels; and miR-200-5p is implicated in the regulation of *MAPKAPK2* and *MAP3K2* expression. *MAP2K2* expression is likely not regulated by the selected miRNAs. At the protein level, our analysis revealed reduced concentrations of all proteins in the culture with LPS compared to the control. Conversely, after the addition of adalimumab to the culture, the levels of MAP3K2 and MAPK9 fell below the detection threshold. Additionally, the expression of DUSP1, DUSP3, and DUSP4 increased, while the expression of MAP2K2 and MAPKAPK2 decreased.

Elevated levels of phosphorylated ERK and p38 MAPK proteins have been detected in psoriatic skin biopsies compared to control samples [58]. This observation may be linked to an altered expression pattern of dual-activity protein phosphatase (DUSP) [59,60], characterized by its ability to dephosphorylate threonine, serine, and tyrosine residues of substrates [60]. The common feature among all members of the DUSP family is the presence of a phosphatase domain containing conserved Asp, Cys, and Arg residues, forming the enzyme’s active site. The distinguishing factor among individual DUSP phosphatases is the presence of the MAP kinase binding (MKB) motif or kinase interacting motif (KIM). Consequently, MAP kinase phosphatases (MKP), considered typical DUSP phosphatases, are identified by the inclusion of the KIM motif, while atypical DUSP lacks this domain in their structure [61] Therefore, DUSP plays a crucial role in regulating MAPK levels [62]. Kjellerup et al. extracted total RNA from cultures of normal epidermal keratinocytes that were stimulated with pro-inflammatory cytokines and psoriatic skin [56]. The researchers demonstrated a significant decrease in *DUSP1* mRNA expression in psoriatic skin lesions compared to nonlesional skin. Additionally, they observed that *DUSP1* mRNA expression does not exhibit an early increase during adalimumab treatment. Therefore, they have proposed that the diminished levels of *DUSP1* may play a role in perpetuating chronic inflammation in psoriasis [56]. Meanwhile, we showed an increase in *DUSP1* mRNA expression regardless of the incubation time of keratinocyte cultures with the drug after prior induction of inflammation, which may be due to a different research model than the one used by Kjellerup et al. [56]. In addition, Zhang et al. demonstrated that DUSP1-deficient mice exhibited impaired immune responses, particularly affecting T lymphocytes. Moreover, the population of Th1 and Th17 phenotype lymphocytes was diminished compared to wild-type mice [63]. Meanwhile, the increase in DUSP1 expression we observed at the mRNA level compared to the control and LPS-stimulated cultures may indicate that miRNA molecules showing affinity for DUSP1 mRNA enhance the expression of the transcript and the protein encoded by it [64–66]. It is also possible that in our experiment, the incubation time of the culture with the drug was insufficient to silence DUSP1 expression. In addition, adalimumab may exert a limited inhibitory effect on transforming growth factor beta (TGF-β) and interleukin-6 (IL-6), both crucial for the conversion of naive T cells into Th17 lymphocyte as a primary source of interleukin-17 (IL-17), which has established effects on psoriasis development [67,68]. Chen et al. noted that the heightened expression of miR-34a not only hampers the proliferation of HaCaT cells but also triggers apoptosis [69], which appears to be a positive outcome of adalimumab’s action. Additionally, Chen et al. propose that miR-34a could be a potential therapeutic target in treating psoriasis [69]. Furthermore, Wang et al. observed an elevated level of miR-34a in the skin of individuals with psoriasis vulgaris [70]. The connection between DUSP1 and miR-34a has been previously identified in the context of osteosarcoma [71] and pulmonary artery smooth muscle cells [72].

As highlighted by Zaba et al., the set of inflammatory genes regulated by etanercept (anti – TNF-α-drug) increases gradually over time [73]. It indicates additional indirect effects of TNF-α on more intricate cellular or molecular circuits. Furthermore, TNF-α serves as a direct activator of nuclear factor-kB, the classical transcription factor for TNF-α. Consequently, genes primarily regulated by this transcription factor would normalize as the activation stimulus is dampened by anti – TNF-α-drug [73]. On the other hand, TNF-α exerts broader downstream or indirect actions. Consequently, TNF-α blockers like adalimumab may regulate various transcription factor pathways over time, and the inhibition of TNF-α might lead to different kinetics in normalizing genes governed by diverse transcription factors across multiple cell types. The involution response could be considered a reverse cascade unfolding over time [73].

Research by Mrowka et al. suggests that silencing DUSP3 expression leads to reduced inflammation, a finding confirmed in a mouse model [74]. Thus, the increase in DUSP3 expression we noted may be due to both the regulatory role of miR-34a against this transcript, as well as the stimulation of biological or cellular processes under the influence of adalimumab in which DUSP3 is involved.

Notably, Signal Transducer and Activator of Transcription (STAT) family proteins function as transcription factors in the cell nucleus, influencing the profile of various proteins with pro- or anti-inflammatory properties [75]. Another member of the DUSP family, DUSP4, plays a crucial role in regulating the Janus kinase/signal transducers and activators of transcription (JAK/STAT) pathway by modulating the phosphorylation and dephosphorylation of STAT5. Elevated DUSP4 levels have been linked to increased STAT5 phosphorylation and activation [76]. Furthermore, Hsiao et al. indicated that silencing DUSP4 expression results in a decreased population of Th17 lymphocytes [77]. Additionally, Johar et al. found overexpression of DUSP4 in patients with systemic lupus erythematosus (SLE) compared to healthy volunteers [78]. In addition, it has to remember that, Dougherty et al. demonstrated that elevated DUSP4 expression mitigates oxidative stress [79]. Correspondingly, Li et al. observed diminished DUSP4 levels in osteoarthritis, and its augmentation hindered the activation of the MAPK pathway, thereby lessening oxidative stress, apoptosis, and the inflammatory response [80]. Wykazaliśmy także, że ekspresja *DUSP4* może być potencjalnie regulowana przez miR-3188 i miR-382. Li et al. demonstrated that elevated miR-3188 expression leads to a decrease in the production of inflammatory cytokines in atherosclerosis [81]. Based on the research performed by Zhou et al. showed that miR-3188 is implicated in the development of reduced cell sensitivity to fulvestrant, an oncological therapy [82]. Zhao et al. recently corroborated these observations, highlighting a significant role of miR-3188 in the emergence of drug resistance during chemotherapeutic treatment. They linked this resistance to the impact of miR-3188 on signal transduction along the mTOR/PI3K/AKT/c-JUN pathway [83]. Therefore, results obtained by us suggest the nuanced role of miRNA molecules, which should not be solely regarded as negative expression regulators but also as enhancers. This is substantiated by the fact that in certain therapeutic strategies, efforts are directed toward diminishing the level of a specific miRNA through anti-miRNA molecules, binding in a complementary manner with the miRNA [84].

Therefore, the increase in DUSP1, DUSP3 and DUSP4 expression that we noted may indicate incomplete inactivation of TNF-α and the signaling pathways it activates. This is all the more understandable since adalimumab exhibits modulatory effects against only those TNF-α molecules that have attached to the anti-TNF-α antibody. This is also confirmed by our previous observations on another *in vitro* and *in vivo* model [85–88]. W przeprowadzonym przez nas badanie wykazaliśmy także na poziomie transkryptomu nadekspresję *MAP2K2, MAPKAPK2, MAP3K2* oraz *MAPK9*, przy czym na poziomie białka stwierdziliśmy obniżenie stężenia białek kodowanych przez wspomniane geny w hodowli HaCaT z adalimumabem w porównaniu zarówno do hodowli kontrolnej i eksponowanej tylko na LPS. Wydaje się, że wzorzec ekspresji wspomnianych genów i kodowanych przez nie białek jest regulowany przez miRNA, w tym *MAPK9*:miR-1275; *MAP3K2/MAPKAPK2(MK2)*:miR-200-5p.

Denninger et al. demonstrated that a reduction in MAPK9 levels exacerbated clinical symptoms associated with the inflammatory response [89]. Also, Bertlesen et al. confirmed that assessing changes in MAPK9 expression profile can be a useful marker for monitoring anti-IL17 therapy in patients with psoriasis [90]. Moreover, Yang et al. observed an intensification of lung inflammation and injury in mice with low MAPK9 levels during sepsis and acute lung injury [91]. Interestingly, miR-1275 has been the focus of numerous studies, indicating its involvement in the regulation of various signaling pathways, such as MAPK, ERK/JNK, Wnt, and phosphoinositide 3-kinase (PI3K)/protein kinase B (AKT). This gene is extensively investigated in cancer, where MAPK9 may act as both a tumor suppressor and inducer, depending on the cancer type [92]. Our study unveiled an downing level of miR-1275 in HaCaT cultures after the addition of adalimumab compared to cultures with LPS alone. This potentially could impact the level of MAPK9 protein, which fell below the detection threshold in the culture exposed to adalimumab.

In addition, in our investigation, we also noted a potential impact of miR-200a-5p on the expression of MAP3K2 and MK2. Huang et al. observed heightened levels of MAP3K2 in cases of acute myocardial infarction. The overexpression of miR-1184, involved in regulating MAP3K2 activity, mitigated hypoxia-induced injury mediated by MAP3K2 [93]. Significantly, Magenta et al. detected increased levels of miR-200a in psoriasis patients [94], aligning with our own observations.

The last of the genes whose expression is potentially regulated by miRNAs is MK2. In the context of the changes in MK2 expression that we noted, of interest is the study by Mavropoulos et al. who demonstrated overexpression of MK2 in psoriasis-lesioned epidermis compared to epidermis without psoriatic lesions [95].

A downstream target within the p38 MAPK signaling cascade is MK2. The p38/MK2 pathway plays a role in various pathological conditions, including inflammatory diseases, metastasis, and resistance mechanisms to anticancer agents. However, the progression of p38 inhibitors to advanced clinical trials has been hindered by their unfavorable systemic side effects. Consequently, MK2 emerged as an alternative target for pathway blockade to circumvent the drawbacks associated with p38 inhibition. Initial ATP-competitive MK2 inhibitors faced challenges such as low solubility, limited cell permeability, and insufficient kinase selectivity [96–99]. Presently, noncompetitive ATP-competitive MK2 inhibitors have been identified to address the selectivity issue. These compounds offer an additional advantage, as they demonstrate effectiveness at lower concentrations compared to ATP-competitive inhibitors. Despite the considerable challenges encountered in developing these inhibitors, the MK2 pathway remains an attractive target for treating inflammation and related diseases. It also holds promise for preventing cancer metastasis and enhancing sensitivity to chemotherapeutics [96–99].

Moreover, as confirmed by the research of Ray et al. inhibition of MK2 activity is associated with a simultaneous reduction in the expression of other pro-inflammatory cytokines, including IL-1β, IL-6 and TNF-α, which in the context of our research and the action of adalimumab is a desirable effect [100]. Only in the case of *MAP2K2*, its expression is not regulated by miRNA molecules. Nevertheless, Krawczyk et al. suggest that *MAP2K2* mRNA may be considered as a molecular marker of response to adalimumab treatment for psoriatic arthritis [101].

In conclusion, our study facilitated the discernment of MAPK-related genes exhibiting altered expression in keratinocytes exposed to LPS and adalimumab, thereby presenting potential significance in psoriasis research. We adeptly predicted candidate miRNAs presumed to orchestrate the regulation of these genes, thereby emerging as plausible markers or therapeutic targets.

The overrepresentation test of MAPK pathway gene products was conducted in the final phase of our study. This analysis categorized the genes encoded by the selected participants into 4 biological processes, 12 molecular functions, and 32 KEGG pathways. These results validate the intricate network of interactions and interdependencies, which pose significant challenges in devising effective therapeutic approaches, particularly in conditions like psoriasis.

Nevertheless, our research is not without inherent constraints. First, the analysis was confined to a single-cell line within an in vitro model. Second, miRNA predictions hinge on bioinformatics databases and lack empirical validation. Third, our scrutiny was restricted to assessing the influence of only one pro-inflammatory agent and a pharmaceutical intervention employed in psoriasis arthritis treatment.

Consequently, the logical and imperative subsequent phase of investigation necessitates an extensive appraisal of MAPK-related genes and miRNAs influencing their expression across diverse cell lines, including normal keratinocytes or in co-culture with leukocytes. Additionally, it is deemed justifiable to scrutinize alterations in the mRNA and miRNA patterns delineated in our study within clinical specimens such as whole blood and serum derived from patients subjected to adalimumab treatment for psoriasis arthritis. Finally, in subsequent stages, our intent is to appraise the impact of other pharmacological agents utilized in psoriasis treatment on the expression of MAPK-related genes and their associated miRNAs. This comprehensive evaluation will also involve elucidating the correlation between their expression and clinical indicators delineating the progression of the disease.

## Data Availability

All data was included in the paper.
